# How close are we to therapies for Sanfilippo disease?

**DOI:** 10.1007/s11011-017-0111-4

**Published:** 2017-09-18

**Authors:** Lidia Gaffke, Karolina Pierzynowska, Ewa Piotrowska, Grzegorz Węgrzyn

**Affiliations:** Department of Molecular Biology, University of Gdańsk, Wita Stwosza 59, 80-308 Gdansk, Poland

**Keywords:** Sanfilippo disease, Neurodegeneration, Lysosomal storage, Enzyme replacement therapy, Gene therapy, Substrate reduction therapy

## Abstract

Sanfilippo disease is one of mucopolysaccharidoses (MPS), a group of lysosomal storage diseases characterized by accumulation of partially degraded glycosaminoglycans (GAGs). It is classified as MPS type III, though it is caused by four different genetic defects, determining subtypes A, B, C and D. In each subtype of MPS III, the primary storage GAG is heparan sulfate (HS), but mutations leading to A, B, C, and D subtypes are located in genes coding for heparan N-sulfatase (the *SGSH* gene), α-N-acetylglucosaminidase (the *NAGLU* gene), acetyl-CoA:α-glucosaminide acetyltransferase (the *HGSNAT* gene), and N-acetylglucosamine-6-sulfatase (the *GNS* gene), respectively. Neurodegenerative changes in the central nervous system (CNS) are major problems in Sanfilippo disease. They cause severe cognitive disabilities and behavioral disturbances. This is the main reason of a current lack of therapeutic options for MPS III patients, while patients from some other MPS types (I, II, IVA, and VI) can be treated with enzyme replacement therapy or bone marrow or hematopoietic stem cell transplantations. Nevertheless, although no therapy is available for Sanfilippo disease now, recent years did bring important breakthroughs in this aspect, and clinical trials are being conducted with enzyme replacement therapy, gene therapy, and substrate reduction therapy. These recent achievements are summarized and discussed in this review.

## Sanfilippo disease – brief description

Mutations in 4 different genes cause mucopolysaccharidosis (MPS) type III, or Sanfilippo disease. These genes code for different enzymes participating in degradation of heparan sulfate (HS), one of glycosaminoglycans (GAG)s. Therefore, if mutations occur in both alleles of one of these genes (each subtype of Sanfilippo disease is inherited in autosomal recessive manner), HS accumulates in lysosomes which is the primary cause of the disease. Depending on dysfunction of particular genes and their products, 4 subtypes of Sanfilippo disease are distinguished, called MPS III A, B, C, and D (OMIM no. 252900, 252,920, 252,930 and 252,940, respectively). They result from mutations in *SGSH* (coding for heparan N-sulfatase), *NAGLU* (coding for α-N-acetylglucosaminidase), *HGSNAT* (coding for acetyl-CoA:α-glucosaminide acetyltransferase), and *GNS* (coding for N-acetylglucosamine-6-sulfatase), respectively. Patho-mechanisms and characteristic features of Sanfilippo disease have been reviewed recently, thus, these articles should be considered for more detailed information (Andrade et al. 2015; Fedele 2015; Jakobkiewicz-Banecka et al. 2016).

Irrespective of the subtype, all persons suffering from Sanfilippo disease develop similar symptoms, though their severity and time of appearance may differ significantly from patient to patient. Unlike other MPS types, where extremely severe somatic symptoms develop, the major clinical problems of MPS III arise from cognitive defects and neurological dysfunctions (although they also occur in some other MPS types, it appears that central nervous system (CNS) dysfunction is the most severe in Sanfilippo disease). The symptoms appear usually at the age of several month to a few years, and include developmental delay, cognitive decline, hyperactivity, sleep disturbances, aggression-like behavior, and seizures. At the late phase of the disease, hyperactivity and anxiety disappear, but patients lose their ability to move, and react poorly to external impulses. The life spam is usually between 2 and 3 decades (for detailed reviews see: Andrade et al. 2015; Fedele 2015; Jakobkiewicz-Banecka et al. 2016).

## Development of therapies for Sanfilippo disease

Because the major clinical problems found in MPS III are due to CNS dysfunctions, development of effective therapy for Sanfilippo disease is extremely difficult. In some other MPS types (I, II, IVA, and VI), where severe somatic symptoms appear, enzyme replacement therapy (ERT) and, hematopoietic (or bone marrow) stem cell transplantation (HSCT) are available, which provide a possibility to manage various problems caused by the disease (Giugliani et al. 2016). However, even if appropriate enzyme is delivered to the blood of MPS III patients, it cannot cross efficiently the blood-brain-barrier, therefore, no significant therapeutic effects can be obtained.

Despite the problems outlined above, in recent years, many excellent reports have been published which described a huge progress in the efforts to find a therapeutic solution for patients suffering from Sanfilippo disease. Three groups of potential therapies can be distinguished: modified ERT (in which the enzyme is either fused to a factor that is able to cross the blood-brain-barrier or administered directly to CNS), gene therapy, and therapies based on small molecules. Importantly, apart from studies on cellular and animal models of MPS III, clinical trials have been started with all these three therapeutic options. Although no such therapy has been registered yet, it may appear that we are quite close to obtain real therapeutic options for Sanfilippo patients in near future. In this review, the above mentioned works will be summarized and discussed, with special emphasis on possibilities of development of effective treatment procedures. We will focus on articles published during last 3 years, as previous works in this filed have already been reviewed (Andrade et al. 2015; Fedele 2015; Jakobkiewicz-Banecka et al. 2016; Sorrentino and Fraldi 2016), whereas this period was especially reach in breakthrough reports.

It is worth to mention that due to specific clinical problems appearing in Sanfilippo disease (see above), it is very difficult to determine unambiguous tests which could be used in clinical trials for assessment of efficacy of tested therapies. This problem is enhanced by the fact that MPS III is a rare disease (prevalence is estimated between 0.3 and 4.1 cases per 100,000 newborns; Valstar et al. 2008), and thus, any clinical trial may involve only relatively small number of patients. Therefore, specific recommendations on clinical trial design for treatment of Sanfilippo disease have been recently established by a group of experts in the field (Ghosh et al. 2017).

## Recent studies on cellular models of MPS III

Cellular models of genetic diseases provide a possibility for effective determination of molecular mechanisms of tested processes and of efficiency of assessed therapuetics. Although studies on such models cannot wholy reflect possible reactions of organisms to tested compounds, they provide a potent tool for either preliminary testing of potential drugs or assessing specific mechanism by which tested compunds act in the cell. Recent reports describing results of experiments with cellular models of Sanfilippo disease provided important data facilitating development of ERT, substrate reduction therapy (SRT) and other therapies based on small molecules for this disorder.

### Enzyme replacement therapy

The mojor problem of ERT for Sanfilippo disease is a lack of possibility of efficient crossing the blood-brain-barrier by the recombinant enzyme. Moreover, an additional problem in MPS IIIB is inadequate mannose 6 phosphorylation and resultant poor uptake of recombinant human α-N-acetylglucosaminidase (rhNAGLU) by cells. In order to overcome the latter drawback, Kan et al. (2014) constructed the rhNAGLU enzyme fused to the fragment of insulin-like growth factor 2 (IGF-II) that is responsible for binding to the IGF-II receptor. Therefore, such a fusion protein should be able to enter the cells through the receptor for IGF-II. Indeed, it was demonstrated that the fusion protein was effectively transported inside MPS IIIB cells. Moreover, it retained the α-N-acetylglucosaminidase activity, as was able to reduce the amount of HS in MPS IIIB fibroblasts to the level observed in control cells (Kan et al. 2014).

These results indicated that fusion of the desired enzyme with the receptor-binding fragment of IGF-II may be effective in overcoming poor enzyme delivery if mannose 6 phosphorylation is inefficient. However, to develop ERT for MPS IIIB, another problem, low efficiency of crossing the blood-brain-barrier, must also be solved.

### Substrate reduction therapy

The rationale of SRT is to reduce the efficiency of synthesis of compounds that cannot be efficiently degraded, thus, establishing a new balance in their metabolism. In this light, SRT for MPS is based on reduction of synthesis of GAGs. It was demonstrated previously that one of possibilities is to silence expression of genes coding for enzymes involved in GAG synthesis (Dziedzic et al. 2010; Kaidonis et al. 2010). In fact, the use of siRNA to silence of *XYLT1, XYLT2, GALTI and GALTII* genes (Dziedzic et al. 2010), and the use of shRNA to silence *EXTL2* and *EXTL3* (Kaidonis et al. 2010) have been demonstrated to be effective in reducing GAG synthesis and decreasing HS storage in MPS IIIA. In the recent report, efficiency of silencing of *EXTL2* and *EXTL3* by siRNA has been shown to result in decreased synthesis rate and lowered accumulation of GAG in MPS IIIC fibroblasts (Canals et al. 2015). Although these results could perhaps be predictable on the basis of previous studies (those by Dziedzic et al. 2010 and Kaidonis et al. 2010), it was important to confirm that the SRT strategy based on gene silencing is effective in various subtypes of Sanfilippo disease. On the other hand, despite encouraging results obtained in cell culture experiments, siRNA-mediated gene silencing remains only putative therapy for MPS III as long as a method for efficient delivery of therapeutic molecules to CNS is not developed.

Another way to reduce synthesis of GAG is inhibition of the epidermal growth factor receptor-dependent signal transduction (Jakobkiewicz-Banecka et al. 2009). This can be achieved by the use of genistein, a natural isoflavone, which is able to partially inhibit GAG production and to significantly reduce GAG storage in various types of MPS (Piotrowska et al. 2006). A kind of SRT, called gene expression-targeted isoflavone therapy (GET IT) and based on the use of genistein, has been developed, including studies on cellular and animal models of MPS IIIA and IIIB, and pilot clinical trials (for a review, see Wegrzyn 2012). Further development of this therapy is described in the subsequent chapter devoted to clinical trials.

In a recent study performed by Fumić et al. (2017), a highly active and purified extract of flavonoids from *Medicago sativa* has been obtained. These authors have confirmed that pure genistein is effective in reduction of GAG levels in MPS III cells (they did not specify what subtypes of Sanfilippo disease were studied), and demonstrated similar (but about 25% lower) activity of the flavonoids’ extract. Therefore, these results provide another proof for efficiency of GET IT in reducing GAG levels in Sanfilippo disease. Further studies might indicate what composition of flavonoids is optimal for the most efficient correction of the disease phenotype.

### Other theapies based on the use of small molecules

Although biosynthesis of coenzyme Q10 (CoQ10) is not affected in MPS cells, it was demonstrated that its basal level is decreased in MPS IIIA fibroblasts (Matalonga et al. 2014). This compoud was used, together with an antioxidant cocktail, to treat MPS IIIA and IIIB fibroblasts. Interestingly, MPS IIIB cells treated with CoQ10 indicated an increase in α-N-acetylglucosaminidase activity while no significant changes in heparan N-sulfatase activity could be observed in analogous experiments with MPS IIIA fibroblasts. Reduction in GAG accumulation was also observed in some lines of MPS IIIA and MPS IIIB fibroblasts, particularly those exhibiting enhanced exocytosis (Matalonga et al. 2014). These results might be encouraging to consider CoQ10 supplementation as a procedure that might support management of Sanfilippo disease.

In MPS IIIC, a large proportion (about 20%) of known mutations affects RNA splicing. These mutations are located at the splice sites, thus, one might consider modification of the splicing process as a possible therapeutic option. To rescue normal RNA processing in cells bearing such mutations, specifically modified U1 snRNAs were used (Matos et al. 2014). In another approach, glucosamine has been employed as a potential molecular chaperone to activate the mutant protein which is a product of a gene bearing mutation resulting in shortening of acetyl-CoA:α-glucosaminide acetyltransferase by 4 amino acids (Matos et al. 2014). Both strategies appeared successful in obtaining partial correction of acetyl-CoA:α-glucosaminide acetyltransferase activity. These results may provide a start point to further work on development of the therapy for Sanfilippo disease patients bearing specific mutations in the splice sites. Nevertheless, efficient delivery of therapeutic RNA molecules to CNS has to be ensured, which might be a challenge in this kind of treatment.

When non-sense mutation causes a disease, a stop-codon read-through strategy might be potentially effective. This strategy is based on the use of compounds that are able to modify ribosome activity in such a way that stop codons are not recognized efficiently. Different compounds, both aminoglycosides (gentamicin and geneticin) and non-aminoglycoside compounds (PTC124, RTC13, RTC14, BZ6 and BZ16) were used to treat cells derived from different lysosomal storage diseases (Gómez-Grau et al. 2015). Although in some other diseases (MPS VI, Nieman-Pick disease type A/B) such a strategy was successful in partial restoring deficient enzyme activity, at least by some tested compounds, no positive effects could be observed in cellular models of MPS IIIB and MPS IIIC. Therefore, this therapeutic strategy appears to be ineffective in Sanfilippo disease, at least when compounds tested by Gómez-Grau et al. (2015) are used.

## Recent studies on animal models of MPS III

Animal models of genetic diseases give an opportunity to test potential therapies which efficacies were confirmed in in vitro studies. This stage of therapy development is devoted to test effects of potential drugs on the whole organism, not only on cells.

### The use of umbilical cord mononuclear cells

It is now generally assumed, after different unsuccessful experimental therapy events, that bone marrow or hematopoietic stem cell transplantations are not effective in treatment of Sanfilippo disease (Giugliani et al. 2016; Jakobkiewicz-Banecka et al. 2016). Nevertheless, recent studies indicated some beneficial effects on the use of human umbilical cord mononuclear cells in the mouse model of MPS IIIB (Willing et al. 2014). The cells were administered intravenously, and if the procedure was repeated every month for a half on year, anxiety-like behavior was corrected. In addition, in the brains of treated mice, ganglioside accumulation and microglial activation were decreased, and proper cytoarchitecture was restored in hippocampus (Willing et al. 2014). These results suggest that administration of healthy umbilical cord mononuclear cells might have some therapeutic potential in Sanfilippo disease, despite former lack of success in the use of other cell-based therapies. This might be supported by results of studies on MPS IIIA mice, in which improvement in CNS functions could be demonstrated when the animals were transplanted with hematopoietic stem cells expressing the *SGSH* gene delivered by modified lentiviral vectors (Sergijenko et al. 2013).

### Enzyme replacement therapy

Since CNS is the primary system affected in Sanfilippo disease, there is a serious problem with the use of ERT, where therapeutic enzyme is delivered intraveously, due to inefficient crossing the blood-brain-barrier by the recombinant protein. Therefore, various efforts have been conducted to overcome this drawback.

One of potential methods to deliver an intravenously administered therapeutic enzyme to the brain is to fuse it to another protein which is capable to cross the blood-brain-barrier. Such approach was used by Boado et al. (2014) who constructed heparan N-sulfatase fused to a monoclonal antibody against the human insulin receptor. Following intravenous injection of the fusion protein in the *Rhesus* monkey, about 1% of the protein could be detected in the brain. The recombinant protein retained its enzymatic activity, as it could reduce GAG storage in MPS IIIA fibroblasts (Boado et al. 2014). Analogous work has been performed with α-N-acetylglucosaminidase and MPS IIIB cells, and very similar results were obtained in studies with the *Rhesus* monkey and fibroblasts (Boado et al. 2016). Therefore, the fusion protein strategy appeared to be potentially effective in various subtypes of Sanfilippo disease. It is worth mentioning that an intravenously administered fusion protein has already been applied in a clinical trial with another MPS type, Hurler syndrome (MPS I), and the treatment was well tolerated; the trial is still ongoing (Giugliani et al. 2017).

Another possibility to deliver the enzyme into the brain is its direct administration to CNS. Recombinant heparan N-sulfatase has been infused to cerebrospinal fluid via the cisterna magna of MPS IIIA dogs (King et al. 2015). Reduction of HS levels were observed in the cerebrospinal fluid, as well as in cerebral cortex. Nevertheless, relatively high dose of the enzyme was required to normalize some disease-related biomarkers (King et al. 2015). In another work, effects of different routes of the enzyme administration into the cerebrospinal fluid have been assessed. MPS IIIA mice were treated with recombinant human heparan N-sulfatase by intrathecal lumbar, cisternal and ventricular administration (Beard et al. 2015). The lumbar infusion gave unsatisfactory results (poor enzyme delivery and a lack of significant GAG level reduction), however, the ventricular route, although the most invasive, was the most effective in decreasing GAG levels and reducing microglial activation (Beard et al. 2015). In subsequent study, the same group has modified the administration procedure in which each animal was implanted with an intra-ventricular cannula connected to subcutaneous mini-osmotic pump by which recombinant human heparan N-sulfatase was infused (King et al. 2016b). This allowed for continuous, low-dose influsion of the enzyme into CNS, however, therapeutic effects consisted in partial reduction of primary HS storage and secondary ganglioside storage in the brain, and small reductions in micro- but not astro-gliosis (King et al. 2016b). On the other hand, this method of administration has been subsequently improved by implantation of subcutaneous mini-osmotic pumps connected to an infusion cannula directed at the right lateral ventricle (King et al. 2016a). In MPS IIIA mice treated in this way with heparan N-sulfatase, HS levels almost normalized and amounts of gangliosides decreased significantly (King et al. 2016a).

A combination of the two methods described above, i.e. construction of the fusion protein and direct administration of the enzyme into CNS, has been applied recently (Aoyagi-Scharber et al. 2017). Human α-N-acetylglucosaminidase has been fused with insulin-like growth factor 2 (which ensures more efficient lysosomal targeting) and administered intracerebroventriculary to MPS IIIB mice. Broad distribution of the fusion protein was observed in the brain, which was accompanied with normalization of HS levels and significant reduction of secondary storage (Aoyagi-Scharber et al. 2017).

In summary, either the fusion protein strategy or direct administration of the enzyme to CNS were found to be able to reduce Sanfilippo disease biomarkers in animal models. The use of the enzyme fused to a peptide ensuring effective delivery to the brain appears to be especially attractive option. However, high enzyme doses are required if administered intravenously. This might be overcome by infusion of the enzyme to CNS, but relatively frequent direct administration of the therapeutic compound into CNS is an invasive procedure that might be potentially problematic if hyperactive and irresponsive to commends children, like those suffering from Sanfilippo disease, should be treated.

### Gene therapy

Gene therapy has been supposed for a long time to be a big hope for patients suffering from genetic diseases. However, many years of studies indicated that there are various and serious problems which must be solved before such a therapy can be effctive. Studies of recent years demonstrated that this may be achievable in animal models of Sanfilippo disease.

The choice of appropriate vector for gene therapy appears to be crucial for further efficacy of the tratment. Heldermon et al. (2013) proposed to administer not one but two different viral vectors carrying the recombinant *NAGLU* gene in order to treat MPS IIIB mice. The adeno-associated virus 2/5 (AAV2/5) and lentivirus were used as vectors, and the combined therapy has been compared to results of treatment with each single viurs. Evidently, the combined therapy was the most efficient. Apart from biochemical and histological improvements, treated animals had a significanly longer life span, and indicated considerable improvement in behavioral tests relative to the control group (Heldermon et al. 2013). Even among AAV vectors, different virus serotypes may give various results when administered to organisms. Therefore, 4 AAV vectors bearing the *NAGLU* gene, derived from AAV5, AAV8, AAV9 and AAVrh10, were tested in MPS IIIB mouse model (Gilkes et al. 2016). Following direct injection of the viruses into CNS, various parameters were assessed, including bio-distribution and efficiency of *NAGLU* transduction. Among tested vectors, AAV8 appeared the best one for treatment of MPS IIIB (Gilkes et al. 2016). Nevertheless, one should consider that preferences in this animal model might potentially differ from those in patients suffering from Sanfilippo disesse.

One of the major problem with gene therapy of neurodegenerative disease is efficient delivery of the therapeutic gene to neurons in the brain. The questions are: whether intravenously administered viruses can infect neurons in the brain, and whether direct administration of vector to the brain is safe? A modified AAV9-derived vector bearing *NAGLU* was used to treat cynomolgus monkeys (Murrey et al. 2014). Viruses have been administered via intravenous injection. The most important results of these experiments were that the procedure is safe and that the enzyme is efficiently delivered to CNS (Murrey et al. 2014). Direct delivery of the AAV9 vectors with *NAGLU* to the cerebrospinal fluid of MPS IIIB mice were performed in the study by Ribera et al. (2015). The GAG level normalized and disease symptoms were significantly improved in treated animals, indicating also safety of the procedure (Ribera et al. 2015). Systemic delivery of another vector, rAAV9-CMV-hNAGLU - constructed to transduce the NAGLU gene, has also been demonstrated to be safe and effective when used to treat MPS IIIB mice (Meadows et al. 2015). More detailed studies with the use of this vector indicated that following intravenous injection of viruses into MPS IIIB mice, near-complete correction of levels of different metabolites could be observed (Fu et al. 2017). These results are particularly encouraging in the light of development of the therapy for Sanfilippo disease patients. On the other hand, thalamic administration of 4 different AAV vectors carrying *NAGLU* indicated that this way of delivery of the thearapeutic gene into the brain of MPS IIIB mouse is of limited efficieny (Gilkes et al. 2015), suggesting that such a strategy would not be effective also in humans.

As can be concluded from the above presented description and discussion, majority of recent studies on animal models of gene therapy for Sanfilippo diseasde focused on the MPS IIIB subtype. Nevertheless two other subtypes were also investigated. MPS IIIA mice were treated with two lentiviral vectors bearing genes coding for murine heparan N-sulfatase and sulfatase modifying factor-1 (McIntyre et al. 2014). Following direct administration to cerebral lateral ventricles, effective expression of the therapeutic enzyme was detected in the brain (the activity was between 0.5- and 4-fold of that found in normal mice), and both biochemical and behavioral improvements were evident (McIntyre et al. 2014). In another study, effects of intraparenchymal administration of the AAVrh10-based vector expressing *SGSH* was tested in the mouse model of MPS IIIA (Winner et al. 2016). Although positive changes in various biochemical markers determined in the brains of treated animals were observed in regions located close to the administration site, no such effects could be observed in regions distant from the injection site or those which were not connected to such a site (Winner et al. 2016). Therefore, to achieve satisfactory efficacy, perhaps relatively many injections would be necessary. This, however, raises questions about safety of the treatment. Finally, very recent work has demonstrated the first attempt to test gene therapy in the novel animal model of MPS IIID (Roca et al. 2017). Following administration of the AAV9-derived vector bearing the *GNS* gene to the cerebrospinal fluid of MPS IIID mice, particularly encouraging results were obtained, including normalization of GAG storage, reduction of neuro-inflammation, and correction of behavior. Moreover, life span of the treated animals was significantly extended relative to untreated MPS IIID mice (Roca et al. 2017).

To summarize recent studies on gene therapy for Sanfilippo disease using animal models, we assess that an enormous progress has been done in the fields of effective administration of viral vectors and efficiency of correction of both biochemical parameters in the brains of treated mice and their behavior. The remaining problem is to choose an optimal method for delivery of viral vectors, considering both effectiveness of this process and safety of the procedure. For the latter issue, parameters must be assessed taking into account symptoms of Sanfilippo disease patients, including hyperactivity, very limited or lack of verbal contact, and increased risk during anesthetic procedures.

## Clinical trials

Although clinical trials for different MPS types are being performed since almost 20 years (for a review see Giugliani et al. 2016), until recently, no such trials were reported for Sanfilippo disease. This was mainly because of the difficulty in development of therapy effective in reducing the primary cause of the disease, GAG storage, in the brain. Fortunately, based on extensive studies performed with the use of cellular and animal models, several clinical trials have been reported or started during recent few years. They include stem cell transplantation, enzyme replacement therapy, substrate reduction therapy and gene therapy.

### Cell transplantation-based therapies

Although previous studies indicated that bone marrow transplanation is not effective in Sanfilippo disease (for a review, see Giugliani et al. 2016; Jakobkiewicz-Banecka et al. 2016), attempts were performed to assess efficacy of cell transplantation-based therapies for this disease. A relatively large study, in which 62 MPS patients participated, has been conduced with the use of hematopoietic cell transplantation (Aldenhoven et al. 2015). The results suggested general safety and relatively high efficacy in alleviation of MPS symptoms. However, among 62 patients taking part in this study, only 2 suffered from Sanfilippo disease. Therefore, regarding effects of hematopoietic cell transplantation in MPS III, the results described by Aldenhoven et al. (2015) remained inconclusive.

In another study, umbilical cord blood-derived stem cell transplantation has been performed in 2 patients suffering from Sanfilippo disease (one from MPS IIIA and another from MPS IIIB). The procedure has been conducted before appearance of the disease symptoms (Welling et al. 2015). Patients were monitored for the period of 5 years. However, neurological deterioration, regression of cognitive skills, and behavioral disturbances developed in both patients, despite successful transplantation (Welling et al. 2015). Therefore, one may conclude that, similarly to bone marrow transplantation, cell transplantation-based therapies are also not effective in Sanfilippo disease.

### Enzyme replacement therapy

Results of studies with cellular and animal models (summarized in preceding chapters) encouraged researchers to start a clinical trial (phase 1/2) with ERT for MPS IIIA. In this open-label study, the recombinant human heparan-N-sulfatase has been administered intrathecally to 12 patients every month, for half a year (Jones et al. 2016). This treatment appeared generally safe, though adverse effects of mild-to-moderate severity were reported in all patients; it is important to note that none of them was directly related to the recombinant enzyme. Despite evident reduction in the HS levels in the cerebrospinal fluid, out of 12 patients, 4 showed a decline in developmental quotient, 6 were stable, and results with remaining 2 were inconclusive (Jones et al. 2016). Therefore, although perhaps acceptable safety profile, this treatment definitely requires further studies before solid conclusions on its efficacy can be drawn. The general problem is to assess effects in a relatively small group of patients (which cannot be overcome in rare disorders, like Sanfilippo disease) that represent very different stage and severity of symptoms. Undoubtedly, recent recommendations on clinical trial design for treatment of Sanfilippo disease patients (Ghosh et al. 2017) should be of particular importance in planning further studies in this field.

### Substrate reduction therapy

A kind of SRT for Sanfilippo disease has been proposed, and tested in cellular and animal models (see preceding chapter). This therapy, based on the use of genistein, a natural isoflavone, works by down-regulation of expression of genes coding for enzymes involved in GAG synthesis. Results of pre-clinical studies with this treatment, called gene expression-targeted isoflavone therapy (GET IT), has been summarized previously (Wegrzyn 2012). Encouraging results of those studies led to tests of safety of high dose (150 mg/kg/day) genistein treatment (only such high dose was sufficiently effective in experiments with the MPS IIIB mouse model) in children. The trial was conducted for 12 months, and a very good safety profile of high dose oral genistein therapy has been reported (Kim et al. 2013). Thus, phase 3, double blinded, randomized, placebo controlled clinical trial of high dose oral genistein aglycone in patients with Sanfilippo disease, is ongoing now (Jakobkiewicz-Banecka et al. 2016; EudraCT number 2013–001479-18; https://www.clinicaltrialsregister.eu/ctr-search/trial/2013-001479-18/GB; reference as on August 16, 2017). Genistein is administered orally for 12 months at the dose of 150 mg/kg/day. Twenty four patients with MPS III (subtypes A, B, and C) are involved. The primary endpoint will be evaluation of a decrease in the level of HS in the cerebrospinal fluid at 52 weeks of the treatment, and secondary endpoints include biochemical tests (urinary GAG excretion and plasma HS levels, neuropsychological measures and actigraphy). According to the protocol, this trial should be finished in 2017, therefore, its results may be available in several months.

Regarding safety of genistein, one should note that some adverse effects were observed in healthy mice when very high doses (500 and 1000 mg/kg) of this isoflavone were used. Particularly, levels of following enzymes were elevated: alanine aminotransferase, aspartate aminotransferase, and alkaline phosphatase. Moreover, degeneration of liver was noted, and oxidative stress parameters were increased (Singh et al. 2014). However, no such effects were observed at lower doses (160 mg/kg/day or less) in wild-type and MPS IIIB mice (Malinowska et al. 2009, 2010). On the other hand, during studies on MPS I mice, decreased body length and femur length, as well as a scrotal hernia and/or scrotal hydrocele, were observed in a fraction of animals treated with genistein at the dose of 160 mg/kg/day (Kingma et al. 2015). Therefore, this isoflavove should be used with caution, especially when administered at high doses.

### Gene therapy

Gene therapy appeared to be a great hope for MPS patients, particularly those suffering from neurodegenerative forms of this disease. One should note that studies on such therapies are being conducted for different MPS types (summarized by Lau and Hemsley 2017). However, in this review, we will focus solely on clinical trials with Sanfilippo disease patients.

The first clinical trial with gene therapy for Sanfilippo disease was reported by Tardieu et al. (2014). AAVrh10-based vector, bearing genes coding for heparan-N-sulfamidase and sulfatase-modifying factor 1, has been injected intracerebrally. Four MPS IIIA patients were involved in this open-label phase 1/2 trial. Despite the highly invasive procedure of the vector administration, the treatment was generally well tolerated, and no recombinant enzyme-related adverse events occurred. Preliminary evaluation of the treatment efficacy was inconclusive, as results varied significantly between patients; for example, brain atrophy stabilized in 2 patients, but proceeded in other, and some improvement in behavior, attention and sleep could be noted in a half of patients (Tardieu et al. 2014). Therefore, no solid conclusions on efficacy of this gene therapy procedure can be reached before more advanced clinical studies are conducted.

Results of a similar clinical trial phase 1/2 with AAV2/5-based vector carrying the *NAGLU* gene have been reported recently (Tardieu et al. 2017). In this open-label study, 7 patients suffering from MPS IIIB participated and received 16 intraparenchymal deposits of the vector mentioned above. Safety profile was acceptable, and neurocognitive development was improved relative to data obtained from description of the natural history of the disease. Importantly, the best results were observed in the youngest patient (Tardieu et al. 2017), suggesting that such a therapy should be started relatively early to be effective. Definitely, further clinical studies are required to assess efficacy of this therapy.

## Concluding remarks

During recent years, many particularly important reports were published in the field of development of therapies for Sanfilippo disease. This information is crucial in the light of current lack of any registered therapeutic procedure for this disorder. There is an outstanding progress in both understanding mechanisms of the disease and developing treatment methods. From experiments on cellular and animal models, as well as from results of the first clinical trials, it appears that enzyme replacement therapy (with either direct administration of the enzyme into CNS or construction of fusion proteins consisting of the desired enzyme and a polypeptide responsible for efficient crossing the blood-brain-barrier), substrate reduction therapy (with the use of genistein as a factor causing decreased level of expression of genes coding for enzymes involved in GAG synthesis, thus slowing down this process), and gene therapy (with vectors administered either intravenously or directly to the central nervous system) are the most advanced therapeutic procedures that are currently tested in clinical trials. One might ask: how close are we to real therapies for Sanfilippo disease? On one hand, initiation of clinical trials may suggest that registration of treatment procedures available for MPS III patients should be possible in a few years. On the other hand, despite impressive results of studies of various therapies on animal models, until now, no spectacular cure was reported in the case of patients suffering from Sanfilippo disease. Perhaps either optimization of currently tested therapeutic procedures or combination of two (or more) different therapies might provide a treatment effective enough to overcome most, if not all, clinical problems occurring in MPS III. Finally, it is worth noting that there is a disproportion in the number of studies conducted on different subtypes of Sanfilippo disease. Definitely, enzyme replacement therapy and gene therapy may be available quicker for subtypes MPS IIIA and IIIB than for others. On the other hand, substrate reduction therapy should be equally effective for all MPS III subtypes.

In conclusion, the current state of published results of studies on therapies for Sanfilippo disease is summarized in Table 1. It is also worth underlining that several clinical trials for MPS III are ongoing now (see: https://www.clinicaltrialsregister.eu/, https://clinicaltrials.gov/, references as on August 14, 2017), and next months and years should provide another portion of excellent articles describing their results.

**Table 1:** Summary of studies conducted on development of therapies for Sanfilippo disease (MPS III) and reported during last 3 years (2014–2017)

MPS III subtype	Models or trials	Therapies investigated in recent years			
		ERT	SRT	Other small molecules	Gene therapy
MPS IIIA	Cellular models	X	X	X	
	Animal models	X			X
	Clinical trials	X	X		X
MPS IIIB	Cellular models	X	X	X	
	Animal models	X	X		X
	Clinical trials		X		X
MPS IIIC	Cellular models		X	X	
	Animal models				
	Clinical trials		X		
MPS IIID	Cellular models				
	Animal models				X
	Clinical trials				

The availability of reports on particular kinds of therapies and models are marked by X

*Abbreviations*: *MPS* mucopolysaccharidosis, *ERT* enzyme replacement therapy, *SRT* substrate reduction therapy
